# Increased Glymphatic System Activity and Hypothalamic Connectivity in Patients With Premenstrual Dysphoric Disorder

**DOI:** 10.1155/da/3641238

**Published:** 2026-04-15

**Authors:** Chengxiang Liu, Xiaowen Xu, Yujia Li, Sirui Chen, Dongmei Liu, Yintao Liu, Jingdong Lu, Peng Liu, Hai Liao

**Affiliations:** ^1^ School of Life Science and Technology, Xidian University, Xi’an, Shaanxi, China, xidian.edu.cn; ^2^ Engineering Research Center of Molecular and Neuro Imaging, Ministry of Education, Xi’an, Shaanxi, China, moe.edu.cn; ^3^ Department of Radiology, Guangxi Hospital Division of The First Affiliated Hospital, Sun Yat-sen University, Nanning, Guangxi, China, sysu.edu.cn; ^4^ Department of Medical Imaging Center, Guangxi Medical University Cancer Hospital, Nanning, Guangxi, China, gxmu.edu.cn

**Keywords:** diffusion tensor imaging analysis along the perivascular space, functional connectivity, glymphatic system, hypothalamus, premenstrual dysphoric disorder

## Abstract

**Objectives:**

Premenstrual dysphoric disorder (PMDD), which is an emotional disorder characterized by symptoms of irritability, depression, anxiety, and sleep disturbances during the luteal phase of the menstrual cycle. Advanced neuroimaging findings provided insights into the neurobiological underpinnings of PMDD. However, the role of the glymphatic system and hypothalamus‐related functional connectivity (FC) remains unclear. We aimed to investigate the abnormalities of glymphatic system and hypothalamus‐related FC in PMDD patients.

**Methods:**

In this study, 23 PMDD patients and 27 healthy controls (HCs) underwent diffusion tensor imaging (DTI) and functional magnetic resonance imaging scan. The DTI along the perivascular space (DTI‐ALPS) indices were used as an indirect evaluation of glymphatic function. Altered hypothalamus‐related FC was detected between PMDD patients and HCs, and machine learning was performed to assess the classification performance of these FC abnormalities in distinguishing PMDD patients from HCs. Leave‐one‐out cross‐validation was used for modal validation. Classification accuracy and area under the receiver operating characteristic (ROC) curve were evaluated. Furthermore, the associations between DTI‐ALPS index, clinical features, and hypothalamus‐seeded FC were explored.

**Results:**

Compared to HCs, PMDD patients exhibited: (1) significantly higher value of left DTI‐ALPS index; (2) increased intrinsic connectivity between the hypothalamus and anterior/middle cingulate cortex (ACC/MCC), middle frontal cortex (MFC), orbitofrontal cortex (OFC), insula, inferior temporal cortex (ITC), inferior parietal lobe (IPL), caudate, lentiform nucleus, and thalamus. The right and mean DTI‐ALPS indices were positively correlated with the hypothalamus‐right ACC/MCC FC in PMDD patients. Significantly positive correlations were observed between the hypothalamus‐right ITC FC and anxiety, depression scores, as well as between the hypothalamus‐bilateral lentiform nucleus FC and severity of symptoms in PMDD patients. Hypothalamus‐related FC showed superior discriminative ability in differentiating PMDD patients from HCs.

**Conclusion:**

The increased glymphatic system activity and hypothalamus‐related FC might be associated with the supersensitive reactivity of emotional processing in PMDD patients. Brain regions primarily involved in the emotional network showed potential group‐discriminative features when comparing PMDD patients to HCs.

**Trial Registration:** Chinese Clinical Trial Registry identification: ChiCTR2000040935

## 1. Introduction

Premenstrual dysphoric disorder (PMDD) is characterized by symptoms of irritability, depression, anxiety, physical discomfort, and disruptions to sleep and circadian rhythms, peaking in the late luteal phase of the menstrual cycle [1, 2]. Besides, PMDD affects the quality of life, work, family, relationships, productivity, and social activity for roughly 3%–8% of women during their menstrual period [2, 3]. The etiology factors of PMDD are complex, involving hormonal changes, genetic predisposition, nutritional deficiencies, diet preferences, environment, social aspects, and neurotransmitters alterations [4, 5]. A comprehensive neurobiological understanding of menstrual reactivity is critical for identifying the neuropathology of PMDD.

Increasing researches indicate that estrogen and progesterone can regulate inflammatory activity in the central nervous system (CNS), and the risk of PMDD is influenced by CNS inflammation [6–8], such as the regulation of the hypothalamic–pituitary–gonadal (HPG) axis, hypothalamic–pituitary–adrenal (HPA) axis, serotonin (5‐HT) system, and gamma‐aminobutyric acidergic (GABAergic) system [4, 8–13]. The interaction was revealed between the HPG axis and HPA axis in PMDD patients [11]. The hypothalamus is the center of the HPA axis, located at the rostroventral to the thalamus, involved in the regulation of cognitive, limbic, behavioral, autonomic, and homeostatic processes [14]. The HPA axis has extensive afferent and efferent connections, and its primary functions are associated with negative emotions and stress (i.e., anxiety) [14, 15]. Aggression is one of the possible outcomes of irritability [1]. It was reported that the higher the irritability, the more aggressively the participants behaved during the task in PMDD patients [16]. Compared to healthy controls (HCs), PMDD patients showed irregularities in the HPA axis and immune function, particularly during the luteal phase [12, 13]. However, it remains unclear how the hypothalamus is involved in emotional regulation and whether the hypothalamus‐related connectivity is altered in PMDD patients.

A “waste clearance” system in the CNS was defined as the glymphatic system, which facilitates the exchange of cerebrospinal fluid and interstitial fluid through perivascular channels and aids in clearing metabolites and toxic wastes from the brain [17]. Notably, the impaired glymphatic system causes the accumulation of metabolic wastes in the brain, further affecting the normal activity of neurons and the efficiency of information processing [18, 19], further involving in a variety of symptoms, including cognitive decline [20], emotion fluctuation [21], or inflammatory response [22]. It was confirmed that glymphatic dysfunction contributed to various neuropsychiatric disorders [20–22]. Therefore, the evaluation of glymphatic system is critical for understanding the underlying neurological mechanisms of PMDD.

Diffusion tensor imaging along the perivascular space (DTI‐ALPS) index provides an important metric of the anatomic substrate for fluid flow in the glymphatic system [23]. This method is a noninvasive, reliable, and promising indicator of glymphatic function in the brain, which has been recognized by researchers and widely used in various diseases [20–25], such as depressive disorder [21, 22], Alzheimer’s disease [23], migraine [24], and Parkinson’s disease [25]. Thus, the DTI‐ALPS method provides an opportunity to noninvasively investigate the glymphatic system in PMDD.

In this study, to address the above issues, we applied (1) DTI‐ALPS to detect the alterations in the glymphatic system; (2) the seed‐based functional connectivity (FC) to explore the changed connectivity between the hypothalamus and the whole brain; (3) machine learning to assess the classification performance of abnormal hypothalamus‐related connectivity in distinguishing PMDD patients from HCs; (4) association analysis between DTI‐ALPS index and hypothalamus‐related FC; (5) association analysis between imaging findings and clinical features.

## 2. Methods

The study was conducted according with the Declaration of Helsinki. Each participant was instructed to the whole experiment procedures and signed informed consent forms. The experiment procedures were approved by the Medicine Ethics Committee of First Affiliated Hospital, Guangxi University of Chinese Medicine (Number 2020‐033‐02).

### 2.1. Participants

This study adopted a prospective case–control design. To exclude organic diseases, baseline MRI sequences of the head and pelvis were performed on each participant before the formal scan. Individual sex hormone levels of progesterone and estradiol were measured in this study. Based on the female physical characteristics and hormone levels, all the MRI scanning were performed in the late luteal phase before menstruation.

In order to quantify premenstrual symptoms, according to Diagnostic and Statistical Manual of Mental Disorders‐5th Edition criteria [26], each participant underwent 2 months of prospective screening and was asked to complete a Daily Rating of Severity of Problems (DRSP) scale [27]. Clinical diagnostic interviews were then organized for each participant, during these interviews, the demographic information of each participant was collected. All patients were individually diagnosed by an experienced associate professor gynecologist.

The inclusion criteria of PMDD patients were satisfied: (1) right‐handedness; (2) age: 18–30 years old; (3) the general menstrual cycle range: 24–35 days; (4) in most menstrual cycles, PMDD occurred in 2 weeks before menstruation; (5) the symptoms were associated with abnormality in daily functioning, and given rise to physical, behavioral, or emotional distress; (6) symptoms were alleviated later on the onset of menses and vacant during most of the mid‐follicular phase of the menstrual period; (7) the symptoms were not merely a worsening of another physical chronic or mental disorders; (8) menstrual‐related periodicity, occurrence during the late luteal phase of cycle (days −7 to −1), and absence during the mid‐follicular phase (days +1 to +7), were documented by repeated observations by the PMDD patients basing on DRSP criterion, and a mean luteal phase score at least 50% greater than that during the follicular phase. More detailed inclusion criteria of PMDD patients were provided in the Supporting Information.

The exclusion criteria for PMDD patients were met: (1) having obviously psychiatric disorders diagnosed in accordance with DSM‐5 [26], such as schizoaffective disorder, delusional mental disorder, organic mental disorder, schizophrenia, psychotic features coordinated, or uncoordinated with mood or bipolar disorder; (2) having a history of physical diseases, brain trauma, severe infections or surgeries, gynecological inflammation, menopausal syndrome, dysmenorrhea, thyroid disease, bilateral oophorectomy or hysterectomy, and cancer or mastopathy; (3) taking any drugs in the past 3 months, such as benzodiazepines, steroid compound (e.g., hormonal intrauterine devices and oral contraceptives) or other psychoactive drugs; (4) currently lactating or pregnant; (5) excessive drinking or smoking; (6) those with contraindications for MRI scanning.

The inclusion criteria of HCs were met: (1) right‐handedness; (2) age between 18 and 30; (3) the regular menstrual cycle range: 24–35 days; (4) none of the menstrual cycle scores met the above diagnostic criteria for PMDD; (5) without taking any drugs in the past 3 months; (6) without contraindications for MRI scanning.

### 2.2. Image Acquisition

MRI data were acquired on a 3.0 T Siemens MRI System (Siemens Medical, Erlangen, Germany). During the scanning, each participant was requested to stay still, keep eyes closed and awake, not to consider anything. Foam pillows were used for minimizing movement between the instrument and each participant’s head. DTI images were then obtained with the following parameters: repetition time (TR) = 6800 ms; echo time (TE) = 93 ms, field of view (FOV) = 240 mm × 240 mm, matrix size = 192 × 192, flip angle (FA) = 90°, slice thickness = 3 mm, 62 directions and gradient values, and *b* = 0 and 1000 s/mm^2^. Resting‐state fMRI data were acquired by a single‐shot gradient‐recalled echo planar imaging (EPI) sequence: TR = 2000 ms; TE = 30 ms; FA = 90°; FOV = 240 mm × 240 mm; matrix = 64 × 64; slice thickness = 5 mm; 31 slices and 180 volumes.

### 2.3. Image Preprocessing

All DTI analyses were performed with FMRIB Software Library (FSL; https://fsl.fmrib.ox.ac.uk/fsl). Preprocessing of DTI included eddy current and head motion artifact correction using the FSL diffusion toolbox. The Brain Extraction Tool was employed to remove non‐brain structures. The DTIFIT program was applied to obtain fractional anisotropy (FA). Diffusivity maps in the three dimensions (*x*‐axis, *y*‐axis, and *z*‐axis diffusivity) were also acquired for each participant.

**Figure 1:**
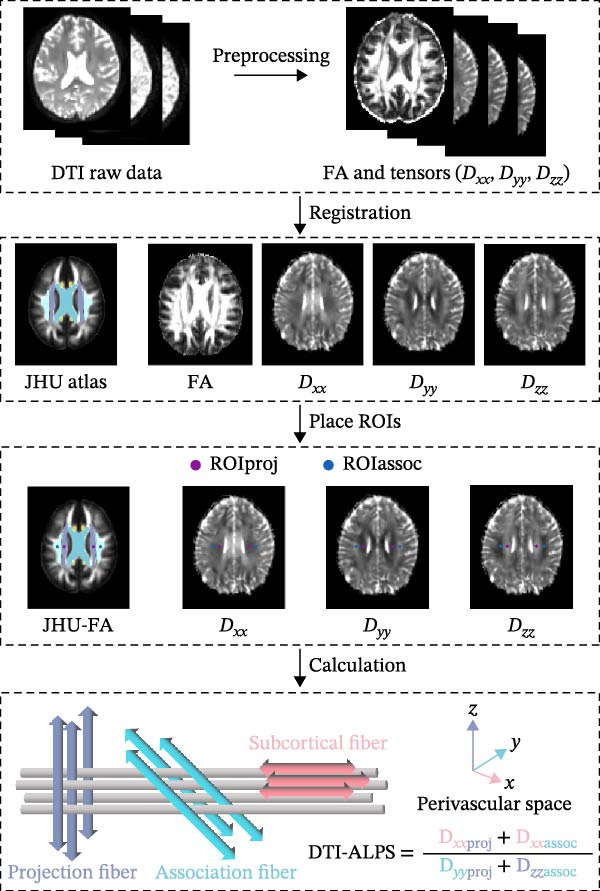
Flow diagram for calculating the DTI‐ALPS index. DTI data were preprocessed to generate FA maps and diffusivity maps (*D*_*xx*_, *D*_*yy*_, *D*_*zz*_), and these images were normalized to a standard template. Four ROIs were placed in the area with projection fibers (projection area), association fibers (association area) to measure diffusivities of the three directions (*x*, *y*, *z*), and then, the DTI‐ALPS index was calculated. DTI‐ALPS, diffusion tensor imaging analysis along the perivascular space; FA, fractional anisotropy; ROI, region of interest.

Individual FA map was co‐registered to the JHU–ICBM–FA template using T1‐weighted image, and the transformation matrix was applied into all the diffusivity maps of *D*_*xx*_, *D*_*yy*_, *D*_*zz*_. All processed images were visually checked to ensure accurate co‐registration. The projection fibers (proj) next to the lateral ventricle ran along the *z*‐axis direction, the association fibers (assoc) ran along the *y*‐axis direction, and the subcortical fibers ran along with the perivascular space in the *x*‐axis direction. On the color‐coded FA map, a 5 mm diameter spherical region of interest (ROI) was placed within the projection fibers and subcortical fibers for each hemisphere (Figure 1). The position of the ROIs for each participant underwent visual inspection, and manual adjustments were carried out by slightly repositioning the position when necessary. In line with previous study [23], the DTI‐ALPS index was calculated as following:

DTI-ALPS index=meanDxxproj,DxxassocmeanDyyproj,Dzzassoc.

The DTI‐ALPS index of the bilateral ROIs was calculated, separately. The average values of the bilateral DTI‐ALPS index were also obtained for subsequent analyses. The calculation process of DTI‐ALPS is shown in Figure 1.

The preprocessing of resting‐state fMRI data in this study was performed using the Data Processing Assistant for Resting‐state fMRI (DPARSF; http://rfmri.org/dparsf) software embedded in Data Processing Analysis of Brain Imaging (DPABI, http://www.rfmri.org/dpabi) toolbox based on Statistical Parametric Mapping 12 (SPM12, http://www.fil.ion.ucl.ac.uk/spm) on MATLAB platform. The primary preprocessing steps included: (1) converting DICOM files to NIFTI images; (2) the first five time points were discarded from each run to remove effects of signal instability; (3) slice timing correction was employed to correct for acquisition delays within functional volumes; (4) realignment (discarded the fMRI data with maximum displacement in any cardinal direction >2.5 mm or head rotation >2.5°); (5) spatial normalization to the Montreal Neurological Institute (MNI) space (EPI template with 3 × 3 × 3 mm^3^ voxel size). Furthermore, nuisance regressions (Friston 24 parameters, cerebrospinal fluid signals, white matter), smooth with 6 mm isotropic Gaussian kernel, removal of linear trends and band‐pass filter (0.01 < *f* < 0.10 Hz) were performed during the preprocessing.

### 2.4. Seed‐Based FC Calculation

The hypothalamus was selected as the ROI of the seed‐to‐voxel FC analysis based on the previous study [28]. For each participant, a FC map was generated by calculating the correlation coefficients between the mean time series of the selected ROI and other voxels’ time series of the brain. The resulting correlation coefficients (*r* values) were converted to *z* values according to Fisher’s *r*‐to‐*z* transformation for further statistical analysis in order to obey a normal distribution. Furthermore, the robustness of hypothalamus‐related FC was validated. As a heterogeneous structure, the distinct subregions of hypothalamus were defined based on one previous study [29], hypothalamic nuclei were assigned to anterior–superior, anterior–inferior, intermediate (also known as “tubular” [30]) and posterior ROIs. The same calculation of FC was performed on hypothalamic subregions.

### 2.5. Statistical Analysis

Demographic characteristics and clinical data of the PMDD patients and HCs were examined by SPSS software (IBM, Armonk, New York). Two‐sample *t*‐test was applied to assess the significant differences of demographic and clinical features between PMDD and HCs. The statistical threshold was set at *p*  < 0.05 for all comparison.

General linear model was applied to detect the between‐group differences in DTI‐ALPS indices (Bonferroni correction, *p*  < 0.05), with age and BMI as covariates.

Two‐sample *t*‐test was performed to explore the between‐group differences in seed‐based FC, with age and BMI as covariates (voxel level *p*  < 0.005 and a cluster level *p*  < 0.05, Gaussian random field correction).

Classification analysis was performed using the MVPANI toolbox [31]. Then, using the hypothalamus‐related FC values of the between‐group differences as input. After that, features with nonzero coefficients remained as the input feature vectors for training classification models. Pattern classification was performed using support vector machine (SVM) and logistic regression (LR). Least Absolute Shrinkage and Selection Operator (LASSO) model was performed to improve the predictive power of classification model and avoid overfitting possibility of the model. Based on the sample size of this study and previous studies [32, 33], leave‐one‐out cross‐validation was used for modal validation. In each repetition, individual’s data was excluded as a test sample, and predictions were made for the remaining individuals’ data. The performance of the classifiers was also evaluated using classification accuracy, receiver operating characteristic (ROC) curve and corresponding area under the curve (AUC). To assess the robustness of the model, 5000 permutation tests were performed (*p*  < 0.05).

Spearman’s correlation analysis was performed to explore the relationships between the DTI‐ALPS indices and hypothalamus‐based FC values in PMDD patients and HCs, respectively (*p*  < 0.05, bootstrap = 1000).

Further, Spearman’s correlation analysis was also applied to explore the relationship between the DTI‐ALPS indices, hypothalamus‐based FC values of the significant brain regions and clinical features (i.e., anxiety, depression, sleep quality, and DRSP score) in PMDD patients and HCs, respectively (*p*  < 0.05, bootstrap = 1000).

## 3. Results

### 3.1. Demographic and Clinical Results

The demographics and clinical features of the participants are summarized in Table 1. PMDD patients had significant differences in Self‐Rating Anxiety Scale (SAS), Self‐Rating Depression Scale (SDS), Pittsburgh Sleep Quality Index (PSQI), and DRSP scores compared to HCs (*p*  < 0.05). No significant difference was found in progesterone and estradiol levels between the PMDD patients and HCs (*p*  > 0.05).

**Table 1:** Demographic and clinical features for PMDD patients and HCs.

Features	Mean ± SD		*p* value
	PMDD (*n* = 23)	HCs (*n* = 27)	
Age (years)	23.70 ± 1.99	23.81 ± 1.67	0.82
BMI	19.25 ± 2.29	20.41 ± 3.86	0.21
Menophania (years)	12.96 ± 1.02	12.96 ± 1.02	0.98
Menstruation (days)	6.30 ± 1.11	5.89 ± 1.19	0.21
Length of menstrual cycle (days)	31.13 ± 3.70	29.52 ± 2.29	0.07
SAS	51.61 ± 10.08	36.56 ± 7.85	<0.001
SDS	56.13 ± 10.63	41.96 ± 9.57	<0.001
DRSP score	70.18 ± 11.90	29.22 ± 5.70	<0.001
PSQI score	8.39 ± 3.04	5.78 ± 1.87	0.001
Progesterone (μg/L)	9.81 ± 5.76	7.51 ± 7.18	0.23
Estradiol (ng/L)	179.04 ± 100.68	154.40 ± 113.87	0.43

*Note:* The *p* value was obtained by two‐sample two‐tailed *t*‐test. PSQI, Pittsburgh sleep inventory.

Abbreviations: BMI, body mass index; DRSP, Daily Rating of Severity of Problems; HCs, healthy controls; PMDD, premenstrual dysphoric disorder; SAS, Self‐Rating Anxiety Scale; SD, standard deviation; SDS, Self‐Rating Depression Scale.

### 3.2. Imaging Results

**Figure 2:**
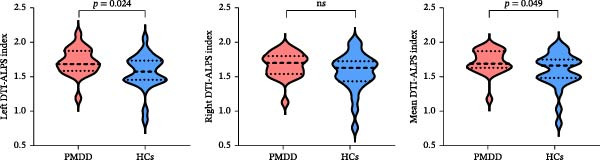
Comparison of bilateral and average DTI‐ALPS indices between PMDD patients and HCs. DTI‐ALPS, diffusion tensor imaging analysis along the perivascular space; HCs, healthy controls; ns, not significant; PMDD, premenstrual dysphoric disorder.

PMDD patients showed significantly higher values of left DTI‐ALPS index relative to HCs (*p* = 0.024; Figure 2). Similarly, PMDD patients exhibited significantly higher values of mean DTI‐ALPS index than HCs (*p* = 0.049; Figure 2).

**Figure 3:**
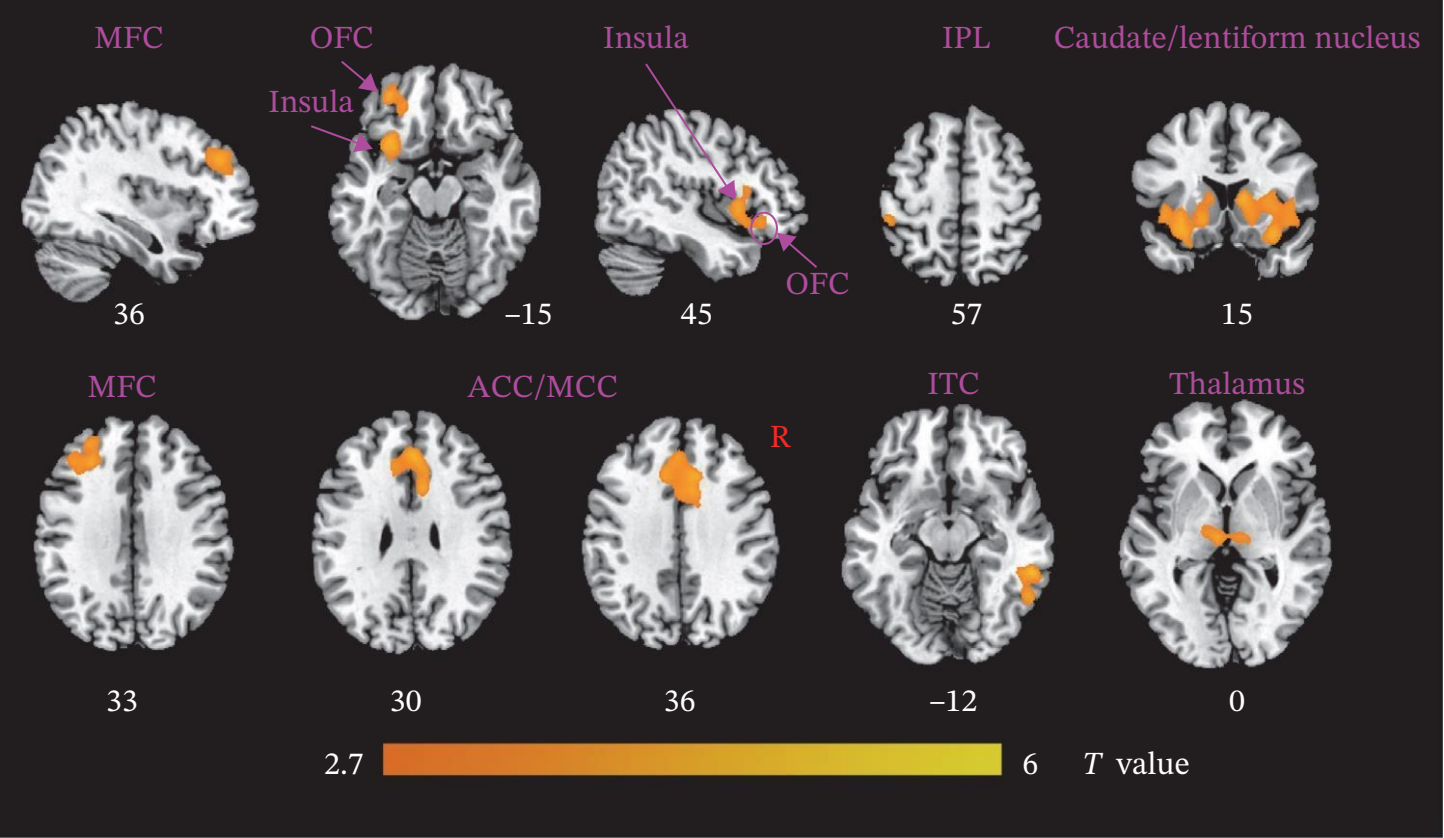
Altered hypothalamus‐related functional connectivity between PMDD patients and HCs. ACC, anterior cingulate cortex; HCs, healthy controls; IPL, inferior parietal lobe; ITC, inferior temporal cortex; MCC, middle cingulate cortex; MFC, middle frontal cortex; OFC, orbitofrontal cortex; PMDD, premenstrual dysphoric disorder.

Compared to HCs, PMDD patients showed significantly increased FC between the hypothalamus and multiple brain regions (Figure 3), including the bilateral middle frontal cortex (MFC), bilateral anterior/middle cingulate cortex (ACC/MCC), bilateral insula, bilateral orbitofrontal cortex (OFC), right inferior temporal cortex (ITC), left inferior parietal lobe (IPL), bilateral thalamus, bilateral caudate, and bilateral lentiform nucleus. No significantly decreased FC was found in PMDD patients compared to HCs. Details of hypothalamic subregions‐related FC are described in Figures S1–S3.

As shown in Figure 4, classification performances showed the superiority of hypothalamus‐related FC features by using SVM (AUC = 0.821, accuracy = 0.780, sensitivity = 0.739, and specificity = 0.815; Figure 4A) and LR (AUC = 0.826, accuracy = 0.780, sensitivity = 0.783, and specificity = 0.778; Figure 4C) models. Permutation tests confirmed that its classification accuracy was significantly higher than the chance level for SVM (*p* = 0.0064; Figure 4B) and LR (*p* = 0.0056; Figure 4D).

Figure 4Classification performance (A) and permutation test results (B) for SVM model; classification performance (C) and permutation test results (D) for LR model. AUC, area under the curve; LR, logistic regression; SVM, support vector machine.

**Figure:**
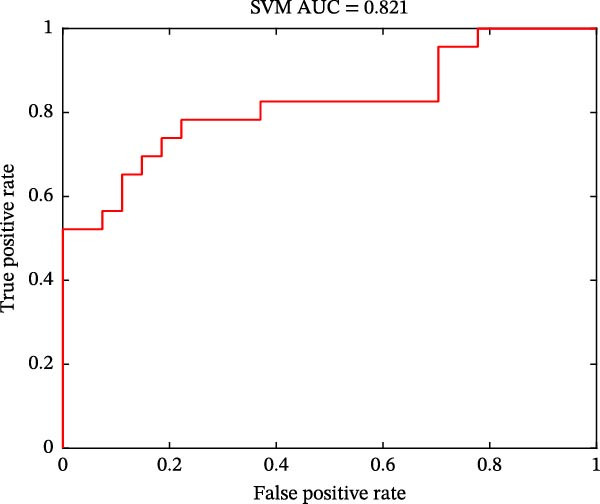
(A)

**Figure:**
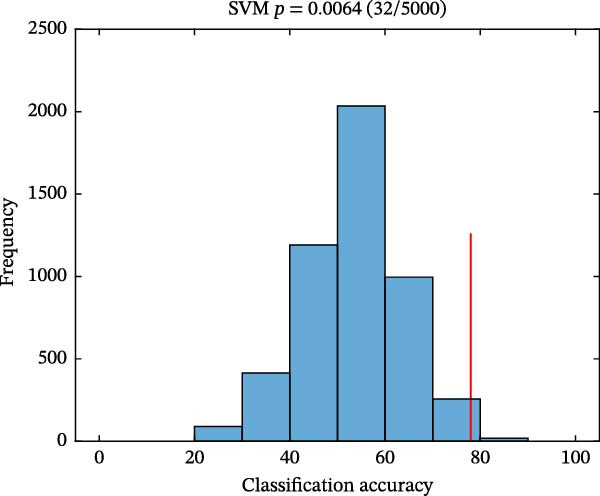
(B)

**Figure:**
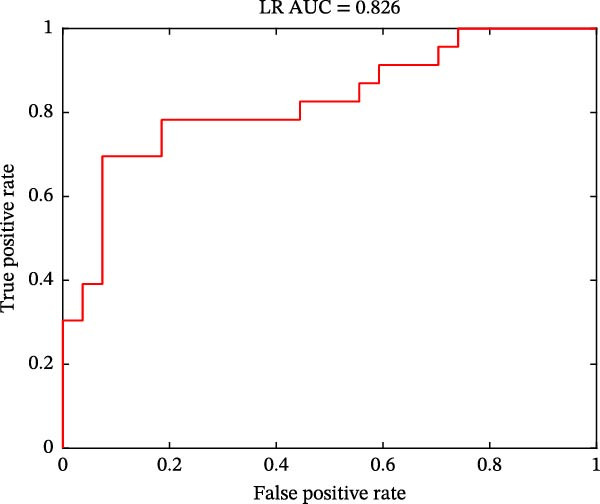
(C)

**Figure:**
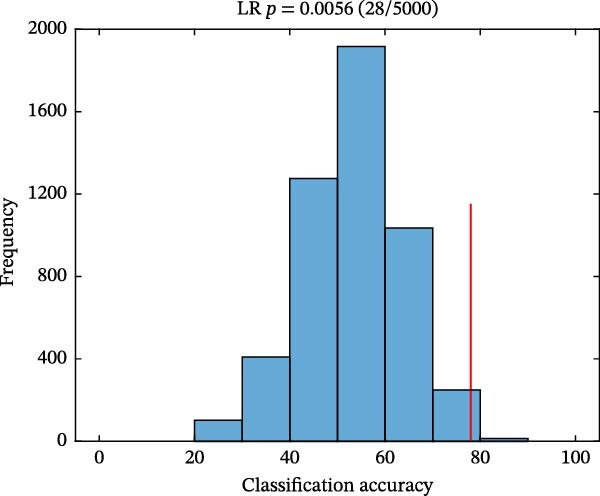
(D)

### 3.3. Correlation Analysis Results

**Figure 5:**
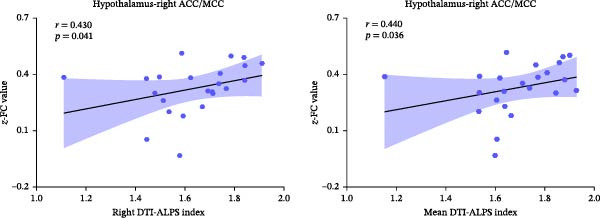
Positive correlation between the right and mean DTI‐ALPS indices and hypothalamus–ACC/MCC connectivity in PMDD patients. ACC, anterior cingulate cortex; DTI‐ALPS, diffusion tensor imaging analysis along the perivascular space; MCC, middle cingulate cortex; PMDD, premenstrual dysphoric disorder.

The right DTI‐ALPS (*r* = 0.430, *p* = 0.041) and mean DTI‐ALPS (*r* = 0.440, *p* = 0.036) indices were significantly positively correlated with the connectivity of hypothalamus‐right ACC/MCC in PMDD patients (Figure 5). However, no significant correlation was found between the DTI‐ALPS indices and hypothalamus‐related FC in HCs.

**Figure 6:**
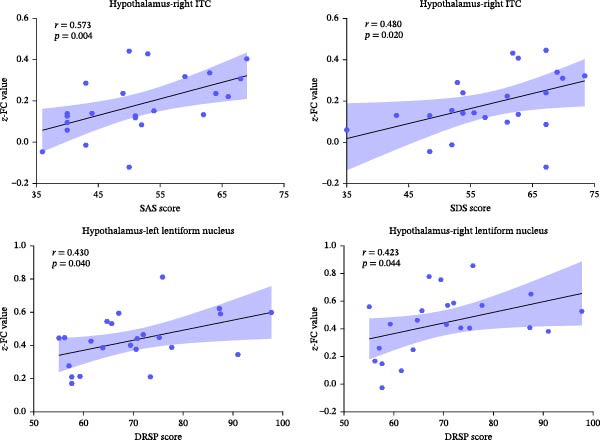
Positive correlations between the hypothalamus‐related functional connectivity (FC) values and clinical features in PMDD patients. DRSP, Daily Rating of Severity of Problems; ITC, inferior temporal cortex; PMDD, premenstrual dysphoric disorder; SAS, Self‐Rating Anxiety Scale; SDS, Self‐Rating Depression Scale.

There were significantly positive correlations (1) between the connectivity of hypothalamus‐right ITC and SAS (*r* = 0.573, *p* = 0.004) and SDS (*r* = 0.480, *p* = 0.020) scores; (2) between the hypothalamus‐left lentiform nucleus FC (*r* = 0.430, *p* = 0.040), hypothalamus‐right lentiform nucleus FC (*r* = 0.423, *p* = 0.044), and DRSP scores (Figure 6). No other significant correlation was found between the DTI‐ALPS indices, hypothalamus‐related FC, and clinical features in HCs.

## 4. Discussion

In this study, we investigated the abnormalities of DTI‐ALPS index, hypothalamus‐related connectivity between PMDD patients and HCs. Furthermore, we explored associations between DTI‐ALPS index, clinical features and hypothalamus‐seeded FC. Compared to HCs, PMDD patients exhibited (1) significantly higher value of DTI‐ALPS index; (2) increased intrinsic connectivity between the hypothalamus and ACC, MCC, MFC, OFC, insula, ITC, IPL, caudate, lentiform nucleus, and thalamus. The right DTI‐ALPS and mean DTI‐ALPS indices were significantly positively correlated with the connectivity of hypothalamus‐right ACC/MCC in PMDD patients. Significantly positive correlations were observed between the connectivity of hypothalamus‐right ITC and SAS, SDS scores, and between the connectivity of hypothalamus‐right lentiform nucleus, hypothalamus‐right lentiform nucleus, and DRSP scores in PMDD patients. The more informative brain regions for the classification power in differentiating PMDD patients from HCs mainly including the ACC/MCC, insula, caudate, lentiform nucleus, thalamus, and ITC.

The significantly higher values of left DTI‐ALPS index in PMDD patents were found compared to HCs, may indicate heightened glymphatic activity. One recent study uncovered an increased DTI‐ALPS index in somatic depression patients, indicating abnormal glymphatic system activity, which is consistent with our findings [21]. Prior studies demonstrated that glymphatic system function, indirectly measured by the DTI‐ALPS index, was strongly correlated with age [34, 35]. The average age of the participants in our study (23.76 years old) is similar to the aforementioned study (24.7 years old), which may be a key factor contributing to the consistent results. Besides, previous study indicated that the basis for the premenstrual onset of depressive symptoms in PMDD patients was cycle‐specific dynamics characterized by increased serotonin uptake before menstruation, followed by a loss of extracellular serotonin [36]. Along this line, depression as one of key symptoms of PMDD patients, the glymphatic system might play a similar role in the pathophysiology of PMDD. The function of the glymphatic system is mediated by aquaporin‐4 and significantly influenced by sleep [34, 37] and endogenous circadian rhythms [38]. It is markedly inhibited when awake but greatly enhanced during sleep, particularly during slow‐wave sleep [39]. Previous studies observed the increased slow‐wave sleep during the luteal phase in PMDD patients compared to HCs [40, 41]. In this study, the PSQI scores of PMDD patients were significantly higher than that of HCs, indicating the PMDD patients had more severer sleep problems in late luteal phase. Clinical studies indicated that PMDD patients exhibited decreased serotonin level [36] and disrupted circadian rhythmicity including blunted, advanced, or delayed nocturnal melatonin secretion [42–44], which may affect glymphatic function. Therefore, we suggested that the increased glymphatic system activity in PMDD patients might be affected by the depressive symptoms and sleep. In our study, the lack of correlation between DTI‐ALPS and SDS/PSQI scores may suggest that the increased depressive symptoms/sleep problems are not directly responsible for the increased activity of the glymphatic system in PMDD patients. However, the effects of depressive symptoms and sleep on glymphatic system function of PMDD need to be investigated further.

Interestingly, the right DTI‐ALPS and mean DTI‐ALPS indices in PMDD patients were positively correlated with the hypothalamus‐right ACC/MCC FC values, indicating that the HPA axis might be involved in the abnormal glymphatic system activity in PMDD.

Notably, PMDD patients demonstrated increased FC between the hypothalamus and multiple regions, mainly including the ACC, MCC, MFC, OFC, IPL, ITC, insula, caudate, lentiform nucleus, and thalamus. Among these, the ACC, OFC, MFC insula, ventral striatum (caudate and lentiform nucleus), thalamus, and IPL constitute a specific network for mood regulation and integrate into a recent model of the emotional brain [45]. PMDD patients characterized by cyclic changes in physiological stress responses and emotional symptoms that align with the menstrual cycle [8]. Perceived stress activates the HPA axis, releasing stress‐related hormones such as corticotropin‐releasing hormone and cortisol to cope with high levels of perceived stress [46]. Abnormal brain functional reactivity of the prefrontal cortex and insula was detected during the emotional stimulation processing in PMDD patients in the late luteal phase [47, 48]. Our study findings that the increased connectivity between hypothalamus and caudate, lentiform nucleus and thalamus were in line with the hyperconnectivity of these regions in PMDD patients [49]. Consistent with the previous study, these increased connectivity between hypothalamus and emotional network might be influenced by fluctuations of ovarian hormones in PMDD patients [50]. The MCC plays a key role in evaluating the importance of visceral stimulation and emotional regulation [51]. The abnormal temporal lobe‐related connectivity reflected the premenstrual syndrome females’ supersensitive reactivity and sensitivity to stress [52, 53]. Moreover, Śliwerski and Bielawska‐Batorowicz [54] confirmed that PMDD patients differentiated from healthy women by their negative cognitive styles. Specifically, PMDD patients tended to present a considerably more negative view of the self, world, and future [54]. Lower reward sensitivity during the luteal phase than that in follicular phase [55]. In this study, PMDD patients had the positive correlations between the hypothalamus–ITC connectivity and SAS and SDS scores, as well as between the hypothalamus–lentiform nucleus connectivity and DRSP score. Positive correlations demonstrated that as the levels of anxiety, depressive symptoms, and severity of disease symptoms increases, hypothalamic connectivity increases as well. Based on these findings, we speculated that the increased hypothalamus‐related connectivity in PMDD patients might be associated with the supersensitive reactivity of emotional processing. The SVM (AUC = 0.821) and LR (AUC = 0.826) results also uncovered the potential group‐discriminative features of hypothalamus‐related FC in distinguishing PMDD patients from HCs.

There are several limitations in this study. First, our study is a cross‐sectional study with a small sample size. In order to reduce the risk of circular feature selection and overfitting, in future studies, a large sample cohort will be needed to validate our findings and the generalization ability of machine learning model. Second, the DTI‐ALPS index indirectly measures the glymphatic function by calculating the diffusivity along the perivenous space at the slice of lateral ventricle body. Thus, the correlation between DTI‐ALPS index and glymphatic activity should be interpreted cautiously. Additional hormonal levels will be appropriately measured in future studies, and further analysis between imaging indices and hormones fluctuations across menstrual cycle will be conducted.

## 5. Conclusions

In summary, this study identified that the abnormalities of glymphatic system, abnormalities of hypothalamus‐related connectivity, might be associated with the supersensitive reactivity of emotional processing in PMDD patients. Additionally, brain regions primarily involved in the emotional network via the HPA axis exhibited potential group‐discriminative features between PMDD patients and HCs. The current study provided new insights into the pathological mechanisms of PMDD patients.

## Author Contributions

Chengxiang Liu contributed to the inception, conception, design and publication of the study, data collection and analysis, drafting of the manuscript, and critical revisions of the manuscript. Xiaowen Xu contributed to the data collection. Yujia Li contributed to the methodological and revisions of the manuscript. Sirui Chen contributed to the data collection. Dongmei Liu, Yintao Liu, and Jingdong Lu contributed to the data analysis. Hai Liao contributed to the conceptualization funding acquisition and project administration. Peng Liu contributed to the inception, conception, design and publication of the study, data collection and analysis, drafting of the manuscript, funding acquisition, project administration, and critical revisions of the manuscript.

## Funding

This study was financially supported by the National Natural Science Foundation of China (Grants 82270696 and 82441050), the Shaanxi Provincial Key Laboratory of Integrated Acupuncture and Medication (Grant KF2219), the Xidian University Specially Funded Project for Interdisciplinary Exploration (Grant TZJHF202521), National Natural Science Foundation of China (Grant 81960570) and the Guangxi Natural Science Foundation (Grant 2017GXNSFBA198095).

## Conflicts of Interest

The authors declare no conflicts of interest.

## Supporting Information

Additional supporting information can be found online in the Supporting Information section.

## Supporting information


**Supporting Information** Detailed inclusion criteria for PMDD patients. Hypothalamic subregions‐related FC results. Figure S1: Altered anterior‐superior (A) and anterior‐inferior (B) hypothalamus‐related FC between PMDD patients and HCs. Figure S2: Altered intermediate hypothalamus‐related FC between PMDD patients and HCs. Figure S3: Altered posterior hypothalamus‐related FC between PMDD patients and HCs.

## Data Availability

The data that support the findings of this study are available from the corresponding author upon reasonable request.

## References

[1] Epperson C. N. , Steiner M. , and Hartlage S. A. , et al.Premenstrual Dysphoric Disorder: Evidence for a New Category for DSM-5, American Journal of Psychiatry. (2012) 169, no. 5, 465–475, 10.1176/appi.ajp.2012.11081302, 2-s2.0-84860515249. 22764360 PMC3462360

[2] Halbreich U. , Borenstein J. , Pearlstein T. , and Kahn L. S. , The Prevalence, Impairment, Impact, and Burden of Premenstrual Dysphoric Disorder (PMS/PMDD), Psychoneuroendocrinology. (2003) 28, 1–23. 10.1016/s0306-4530(03)00098-2 12892987

[3] Hodgetts S. , Kinghorn A. , and Aperribai L. , Examining the Impact of Premenstrual Dysphoric Disorder (PMDD) on Life and Relationship Quality: An Online Cross-Sectional Survey Study, PLOS ONE. (2025) 20, no. 4, 10.1371/journal.pone.0322314, e0322314. 40267093 PMC12017513

[4] Keijser R. , Hysaj E. , Opatowski M. , Yang Y. , and Lu D. , Premenstrual Dysphoric Disorder: Etiology, Risk Factors and Biomarkers, Handbook of the Biology and Pathology of Mental Disorders, 2025, Springer Nature, 1–26.

[5] Erika E.-C. and López-Rubalcava C. , Can Animal Models Resemble a Premenstrual Dysphoric Condition?, Frontiers in Neuroendocrinology. (2022) 66, 10.1016/j.yfrne.2022.101007, 101007. 35623450

[6] Bannister E. , There is Increasing Evidence to Suggest That Brain Inflammation Could Play a Key Role in the Aetiology of Psychiatric Illness. Could Inflammation be a Cause of the Premenstrual Syndromes PMS and PMDD?, Post Reproductive Health. (2019) 25, no. 3, 157–161, 10.1177/2053369119875386, 2-s2.0-85073616443. 31630609

[7] Fedotcheva T. A. , Fedotcheva N. I. , and Shimanovsky N. L. , Progesterone as an Anti-Inflammatory Drug and Immunomodulator: New Aspects in Hormonal Regulation of the Inflammation, Biomolecules. (2022) 12, no. 9, 10.3390/biom12091299, 1299. 36139138 PMC9496164

[8] Cheng M. , Jiang Z. , and Yang J. , et al.The Role of the Neuroinflammation and Stressors in Premenstrual Syndrome/Premenstrual Dysphoric Disorder: A Review, Frontiers in Endocrinology. (2025) 16, 10.3389/fendo.2025.1561848, 1561848. 40225329 PMC11985436

[9] Hantsoo L. and Epperson C. N. , Allopregnanolone in Premenstrual Dysphoric Disorder (PMDD): Evidence for Dysregulated Sensitivity to GABA-A Receptor Modulating Neuroactive Steroids Across the Menstrual Cycle, Neurobiology of Stress. (2020) 12, 10.1016/j.ynstr.2020.100213, 100213. 32435664 PMC7231988

[10] Hantsoo L. and Epperson C. N. , Premenstrual Dysphoric Disorder: Epidemiology and Treatment, Current Psychiatry Reports. (2015) 17, no. 11, 10.1007/s11920-015-0628-3, 2-s2.0-84942116093. PMC4890701 26377947

[11] Beddig T. , Reinhard I. , and Kuehner C. , Stress, Mood, and Cortisol During Daily Life in Women With Premenstrual Dysphoric Disorder (PMDD), Psychoneuroendocrinology. (2019) 109, 10.1016/j.psyneuen.2019.104372, 2-s2.0-85069825759, 104372. 31357135

[12] Hantsoo L. , Jagodnik K. M. , and Novick A. M. , et al.The Role of the Hypothalamic-Pituitary-Adrenal Axis in Depression Across the Female Reproductive Lifecycle: Current Knowledge and Future Directions, Frontiers in Endocrinology. (2023) 14, 10.3389/fendo.2023.1295261, 1295261. 38149098 PMC10750128

[13] Barone J. C. , Ho A. , and Osborne L. M. , et al.Luteal Phase Sertraline Treatment of Premenstrual Dysphoric Disorder (PMDD): Effects on Markers of Hypothalamic Pituitary Adrenal (HPA) Axis Activation and Inflammation, Psychoneuroendocrinology. (2024) 169, 10.1016/j.psyneuen.2024.107145, 107145. 39096755 PMC11381144

[14] Leistner C. and Menke A. , Hypothalamic-Pituitary-Adrenal Axis and Stress, Handbook of Clinical Neurology. (2020) 175, 55–64, 10.1016/B978-0-444-64123-6.00004-7. 33008543

[15] Smith S. M. and Vale W. W. , The Role of the Hypothalamic-Pituitary-Adrenal Axis in Neuroendocrine Responses to Stress, Dialogues in Clinical Neuroscience. (2006) 8, no. 4, 383–395, 10.31887/DCNS.2006.8.4/ssmith. 17290797 PMC3181830

[16] Kaltsouni E. , Fisher P. M. , and Dubol M. , et al.Brain Reactivity During Aggressive Response in Women With Premenstrual Dysphoric Disorder Treated With a Selective Progesterone Receptor Modulator, Neuropsychopharmacology. (2021) 46, no. 8, 1460–1467, 10.1038/s41386-021-01010-9. 33927343 PMC8209206

[17] Iliff J. J. , Wang M. , and Liao Y. , et al.A Paravascular Pathway Facilitates CSF Flow Through the Brain Parenchyma and the Clearance of Interstitial Solutes, Including Amyloid β, Science Translational Medicine. (2012) 4, no. 147, 10.1126/scitranslmed.3003748, 2-s2.0-84865123660, 147ra111. PMC3551275 22896675

[18] Yi T. , Gao P. , Zhu T. , Yin H. , and Jin S. , Glymphatic System Dysfunction: A Novel Mediator of Sleep Disorders and Headaches, Frontiers in Neurology. (2022) 13, 10.3389/fneur.2022.885020, 885020. 35665055 PMC9160458

[19] Cai Y. , Zhang Y. , and Leng S. , et al.The Relationship Between Inflammation, Impaired Glymphatic System, and Neurodegenerative Disorders: A Vicious Cycle, Neurobiology of Disease. (2024) 192, 10.1016/j.nbd.2024.106426, 106426. 38331353

[20] Ran L. , Fang Y. , and Cheng C. , et al.Genome-Wide and Phenome-Wide Studies Provided Insights Into Brain Glymphatic System Function and Its Clinical Associations, Science Advances. (2025) 11, no. 3, 10.1126/sciadv.adr4606, eadr4606. 39823331 PMC11740961

[21] Deng Z. , Wang W. , and Nie Z. , et al.Increased Glymphatic System Activity and Thalamic Vulnerability in Drug-Naive Somatic Depression: Evidenced by DTI-ALPS Index, NeuroImage: Clinical. (2025) 46, 10.1016/j.nicl.2025.103769, 103769. 40120532 PMC11998321

[22] Gong W. , Zhai Q. , and Wang Y. , et al.Glymphatic Function and Choroid Plexus Volume is Associated With Systemic Inflammation and Oxidative Stress in Major Depressive Disorder, Brain, Behavior, and Immunity. (2025) 128, 266–275, 10.1016/j.bbi.2025.04.008. 40220922

[23] Taoka T. , Masutani Y. , and Kawai H. , et al.Evaluation of Glymphatic System Activity With the Diffusion MR Technique: Diffusion Tensor Image Analysis Along the Perivascular Space (DTI-ALPS) in Alzheimer’s Disease Cases, Japanese Journal of Radiology. (2017) 35, no. 4, 172–178, 10.1007/s11604-017-0617-z, 2-s2.0-85012937721. 28197821

[24] Zhang X. , Wang W. , and Bai X. , et al.Increased Glymphatic System Activity in Migraine Chronification by Diffusion Tensor Image Analysis Along the Perivascular Space, The Journal of Headache and Pain. (2023) 24, no. 1, 10.1186/s10194-023-01673-3. PMC10626803 37926843

[25] Dai X. , Zhang Y. , and Fu C. , et al.Investigating Glymphatic Function and Bed Nucleus of the Stria Terminalis-Based Functional Connectivity in Parkinson’s Disease With and Without Depression, npj Parkinson’s Disease. (2025) 11, no. 1, 10.1038/s41531-025-00985-2, 129. PMC12084346 40379669

[26] American Psychiatric Association , Diagnostic and Statistical Manual of Mental Disorders, 2013, 5th edition, American Psychiatric Press.

[27] Endicott J. , Nee J. , and Harrison W. , Daily Record of Severity of Problems (DRSP): Reliability and Validity, Archives of Women’s Mental Health. (2006) 9, no. 1, 41–49, 10.1007/s00737-005-0103-y, 2-s2.0-30644476862. 16172836

[28] Amiel Castro R. T. , Ehlert U. , and Fischer S. , Variation in Genes and Hormones of the Hypothalamic-Pituitary-Ovarian Axis in Female Mood Disorders—A Systematic Review and Meta-Analysis, Frontiers in Neuroendocrinology. (2021) 62, 10.1016/j.yfrne.2021.100929, 100929. 34171352

[29] Spindler M. , Özyurt J. , and Thiel C. M. , Automated Diffusion-Based Parcellation of the Hypothalamus Reveals Subunit-Specific Associations With Obesity, Scientific Reports. (2020) 10, no. 1, 10.1038/s41598-020-79289-9, 22238. 33335266 PMC7747731

[30] Billot B. , Bocchetta M. , Todd E. , Dalca A. V. , Rohrer J. D. , and Iglesias J. E. , Automated Segmentation of the Hypothalamus and Associated Subunits in Brain MRI, NeuroImage. (2020) 223, 10.1016/j.neuroimage.2020.117287, 117287. 32853816 PMC8417769

[31] Peng Y. , Zhang X. , and Li Y. , et al.MVPANI: A Toolkit With Friendly Graphical User Interface for Multivariate Pattern Analysis of Neuroimaging Data, Frontiers in Neuroscience. (2020) 14, 10.3389/fnins.2020.00545, 545. 32742251 PMC7364177

[32] Geroldinger A. , Lusa L. , Nold M. , and Heinze G. , Leave-One-Out Cross-Validation, Penalization, and Differential Bias of Some Prediction Model Performance Measures—A Simulation Study, Diagnostic and Prognostic Research. (2023) 7, no. 1, 10.1186/s41512-023-00146-0. PMC10152625 37127679

[33] Adin A. , Krainski E. T. , Lenzi A. , Liu Z. , Martínez-Minaya J. D. , and Rue H. , Automatic Cross-Validation in Structured Models: Is It Time to Leave Out Leave-One-Out?, Spatial Statistics. (2024) 62, 10.1016/j.spasta.2024.100843, 100843.

[34] Clark O. , Delgado-Sanchez A. , Cullell N. , Correa S. A. L. , Krupinski J. , and Ray N. , Diffusion Tensor Imaging Analysis Along the Perivascular Space in the UK Biobank, Sleep Medicine. (2024) 119, 399–405, 10.1016/j.sleep.2024.05.007. 38772221

[35] Hsiao W.-C. , Chang H.-I. , and Hsu S.-W. , et al.Association of Cognition and Brain Reserve in Aging and Glymphatic Function Using Diffusion Tensor Image-Along the Perivascular Space (DTI-ALPS), Neuroscience. (2023) 524, 11–20, 10.1016/j.neuroscience.2023.04.004. 37030632

[36] Sacher J. , Zsido R. G. , and Barth C. , et al.Increase in Serotonin Transporter Binding in Patients With Premenstrual Dysphoric Disorder Across the Menstrual Cycle: A Case-Control Longitudinal Neuroreceptor Ligand Positron Emission Tomography Imaging Study, Biological Psychiatry. (2023) 93, no. 12, 1081–1088, 10.1016/j.biopsych.2022.12.023. 36997451

[37] Ma J. , Chen M. , and Liu G.-H. , et al.Effects of Sleep on the Glymphatic Functioning and Multimodal Human Brain Network Affecting Memory in Older Adults, Molecular Psychiatry. (2025) 30, no. 5, 1717–1729, 10.1038/s41380-024-02778-0. 39397082 PMC12014484

[38] Hablitz L. M. , Plá V. , and Giannetto M. , et al.Circadian Control of Brain Glymphatic and Lymphatic Fluid Flow, Nature Communications. (2020) 11, no. 1, 10.1038/s41467-020-18115-2, 4411. PMC7468152 32879313

[39] Xie L. , Kang H. , and Xu Q. , et al.Sleep Drives Metabolite Clearance From the Adult Brain, Science. (2013) 342, no. 6156, 373–377, 10.1126/science.1241224, 2-s2.0-84885775321. 24136970 PMC3880190

[40] Shechter A. , Lespérance P. , Ng Ying Kin N. M. K. , and Boivin D. B. , Nocturnal Polysomnographic Sleep Across the Menstrual Cycle in Premenstrual Dysphoric Disorder, Sleep Medicine. (2012) 13, no. 8, 1071–1078, 10.1016/j.sleep.2012.05.012, 2-s2.0-84865287411. 22749440

[41] Baker F. , Sassoon S. , and Kahan T. , et al.Perceived Poor Sleep Quality in the Absence of Polysomnographic Sleep Disturbance in Women With Severe Premenstrual Syndrome, Journal of Sleep Research. (2012) 21, no. 5, 535–545, 10.1111/j.1365-2869.2012.01007.x, 2-s2.0-84866764698. 22417163 PMC3376683

[42] Parry B. L. , Meliska C. J. , and Sorenson D. L. , et al.Increased Sensitivity to Light-Induced Melatonin Suppression in Premenstrual Dysphoric Disorder, Chronobiology International. (2010) 27, no. 7, 1438–1453, 10.3109/07420528.2010.503331, 2-s2.0-77956221162. 20795885 PMC3038841

[43] Nexha A. , Caropreso L. , de Azevedo Cardoso T. , Suh J. S. , Tonon A. , and Frey B. N. , Biological Rhythms in Premenstrual Syndrome and Premenstrual Dysphoric Disorder: A Systematic Review, BMC Women’s Health. (2024) 24, no. 1, 10.1186/s12905-024-03395-3, 551. 39375682 PMC11457342

[44] Shechter A. and Boivin D. B. , Hormones, and Circadian Rhythms Throughout the Menstrual Cycle in Healthy Women and Women With Premenstrual Dysphoric Disorder, International Journal of Endocrinology. (2010) 2010, 17, 10.1155/2010/259345, 2-s2.0-77951066991, 259345. 20145718 PMC2817387

[45] Pessoa L. , A Network Model of the Emotional Brain, Trends in Cognitive Sciences. (2017) 21, no. 5, 357–371, 10.1016/j.tics.2017.03.002, 2-s2.0-85016713792. 28363681 PMC5534266

[46] Russell G. and Lightman S. , The Human Stress Response, Nature Reviews Endocrinology. (2019) 15, no. 9, 525–534, 10.1038/s41574-019-0228-0, 2-s2.0-85068269085. 31249398

[47] Comasco E. , Hahn A. , and Ganger S. , et al.Emotional Fronto-Cingulate Cortex Activation and Brain Derived Neurotrophic Factor Polymorphism in Premenstrual Dysphoric Disorder, Human Brain Mapping. (2014) 35, no. 9, 4450–4458, 10.1002/hbm.22486, 2-s2.0-84904468863. 24615932 PMC4107029

[48] Gingnell M. , Bannbers E. , Wikström J. , Fredrikson M. , and Sundström-Poromaa I. , Premenstrual Dysphoric Disorder and Prefrontal Reactivity During Anticipation of Emotional Stimuli, European Neuropsychopharmacology. (2013) 23, no. 11, 1474–1483, 10.1016/j.euroneuro.2013.08.002, 2-s2.0-84886795286. 24001875

[49] Dan R. , Reuveni I. , and Canetti L. , et al.Trait-Related Changes in Brain Network Topology in Premenstrual Dysphoric Disorder, Hormones and Behavior. (2020) 124, 10.1016/j.yhbeh.2020.104782, 104782.. 32470339

[50] Dubol M. , Epperson C. N. , and Sacher J. , et al.Neuroimaging the Menstrual Cycle: A Multimodal Systematic Review, Frontiers in Neuroendocrinology. (2021) 60, 10.1016/j.yfrne.2020.100878, 100878. 33098847

[51] Elsenbruch S. , Rosenberger C. , Enck P. , Forsting M. , Schedlowski M. , and Gizewski E. R. , Affective Disturbances Modulate the Neural Processing of Visceral Pain Stimuli in Irritable Bowel Syndrome: An fMRI Study, Gut. (2010) 59, no. 4, 489–495, 10.1136/gut.2008.175000, 2-s2.0-77950804750. 19651629

[52] Liu Q. , Li R. , Zhou R. , Li J. , Gu Q. , and Qiu J. , Abnormal Resting-State Connectivity at Functional MRI in Women With Premenstrual Syndrome, PLOS ONE. (2015) 10, no. 9, 10.1371/journal.pone.0136029, 2-s2.0-84943338769, e0136029. 26325510 PMC4556707

[53] Liao H. , Duan G. , and Liu P. , et al.Altered Fractional Amplitude of Low Frequency Fluctuation in Premenstrual Syndrome: A Resting State fMRI Study, Journal of Affective Disorders. (2017) 218, 41–48, 10.1016/j.jad.2017.04.045, 2-s2.0-85018909384. 28458114

[54] Śliwerski A. and Bielawska-Batorowicz E. , Negative Cognitive Styles as Risk Factors for the Occurrence of PMS and PMDD, Journal of Reproductive and Infant Psychology. (2019) 37, no. 3, 322–337, 10.1080/02646838.2018.1543943, 2-s2.0-85057218115. 30468400

[55] Sakaki M. and Mather M. , How Reward and Emotional Stimuli Induce Different Reactions Across the Menstrual Cycle, Social and Personality Psychology Compass. (2012) 6, no. 1, 1–17, 10.1111/j.1751-9004.2011.00415.x, 2-s2.0-84855311856. 22737180 PMC3380631

