# Supplementary material for: Increased Glymphatic System Activity and Hypothalamic Connectivity in Patients With Premenstrual Dysphoric Disorder
Source: Depress Anxiety. 2026 Apr 15;2026:3641238. doi: 10.1155/da/3641238 (PMC13080338; doi:10.1155/da/3641238)

**Supplementary materials**

**Inclusion criteria**

The detailed inclusion criteria of PMDD patients were satisfied: in most menstrual cycles during the past year, five (or more) of the following symptoms occurred during the final week before the onset of menses, started to improve within a few days after the onset of menses, and were minimal or absent in the week post menses, with at least one of the symptoms being either (a), (b), (c), or (d): (a) marked affective lability (e.g., mood swings; feeling suddenly sad or tearful or increased sensitivity to rejection); (b) marked irritability or anger or increased interpersonal conflicts; (c) markedly depressed mood, feelings of hopelessness, or self-deprecating thoughts; (d) marked anxiety, tension, feelings of being "keyed up" or "on edge"; (e) decreased interest in usual activities (e.g., work, school, friends, hobbies); (f) subjective sense of difficulty in concentration; (g) lethargy, easy fatigability, or marked lack of energy; (h) marked change in appetite, overeating, or specific food cravings; (i) hypersomnia or insomnia; (j) a subjective sense of being overwhelmed or out of control; (k) other physical symptoms such as breast tenderness or swelling, joint or muscle pain, a sensation of "bloating", weight gain.

**Hypothalamic subregions-related FC results**

Compared to HCs, PMDD patients showed significantly increased FC: 1) between the anterior-superior hypothalamus and inferior/middle frontal cortex (IFC/MFC) (**Figure S1A**); 2) between the anterior-inferior hypothalamus and insula, supplementary motor area, inferior temporal cortex (ITC), thalamus, caudate and lentiform nucleus (**Figure S1B**); 3) between the intermediate hypothalamus and medial prefrontal cortex (mPFC), anterior/middle cingulate cortex (ACC/MCC), orbitofrontal cortex (OFC), insula, middle frontal cortex (MFC), ITC, caudate and lentiform nucleus (**Figure S2**); and 4) between the posterior hypothalamus and MFC, superior frontal cortex (SFC), postcentral gyrus (PostCG) and ITC (**Figure S3**).

**Figure S1:** Altered anterior-superior (A) and anterior-inferior (B) hypothalamus-related FC between PMDD patients and HCs. **Abbreviations:** FC, functional connectivity; PMDD, premenstrual dysphoric disorder; HCs, healthy controls; IFC, inferior frontal cortex; MFC, middle frontal cortex; SMA, supplementary motor area; ITC, inferior temporal cortex.


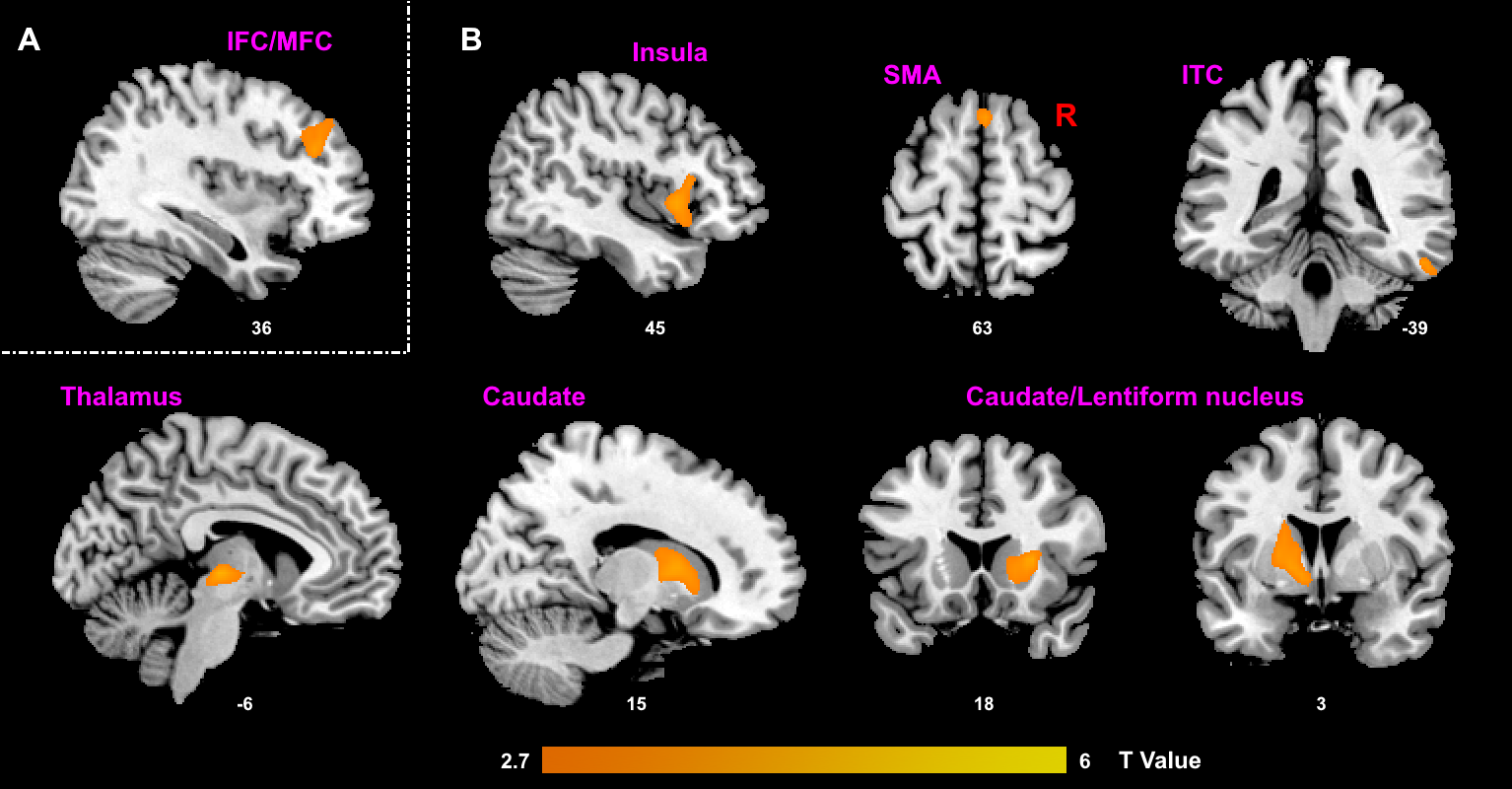


**Figure S2:** Altered intermediate hypothalamus-related FC between PMDD patients and HCs. **Abbreviations:** FC, functional connectivity; PMDD, premenstrual dysphoric disorder; HCs, healthy controls; ACC, anterior cingulate cortex; MCC, middle cingulate cortex; OFC, orbitofrontal cortex; MFC, middle frontal cortex; ITC, inferior temporal cortex.


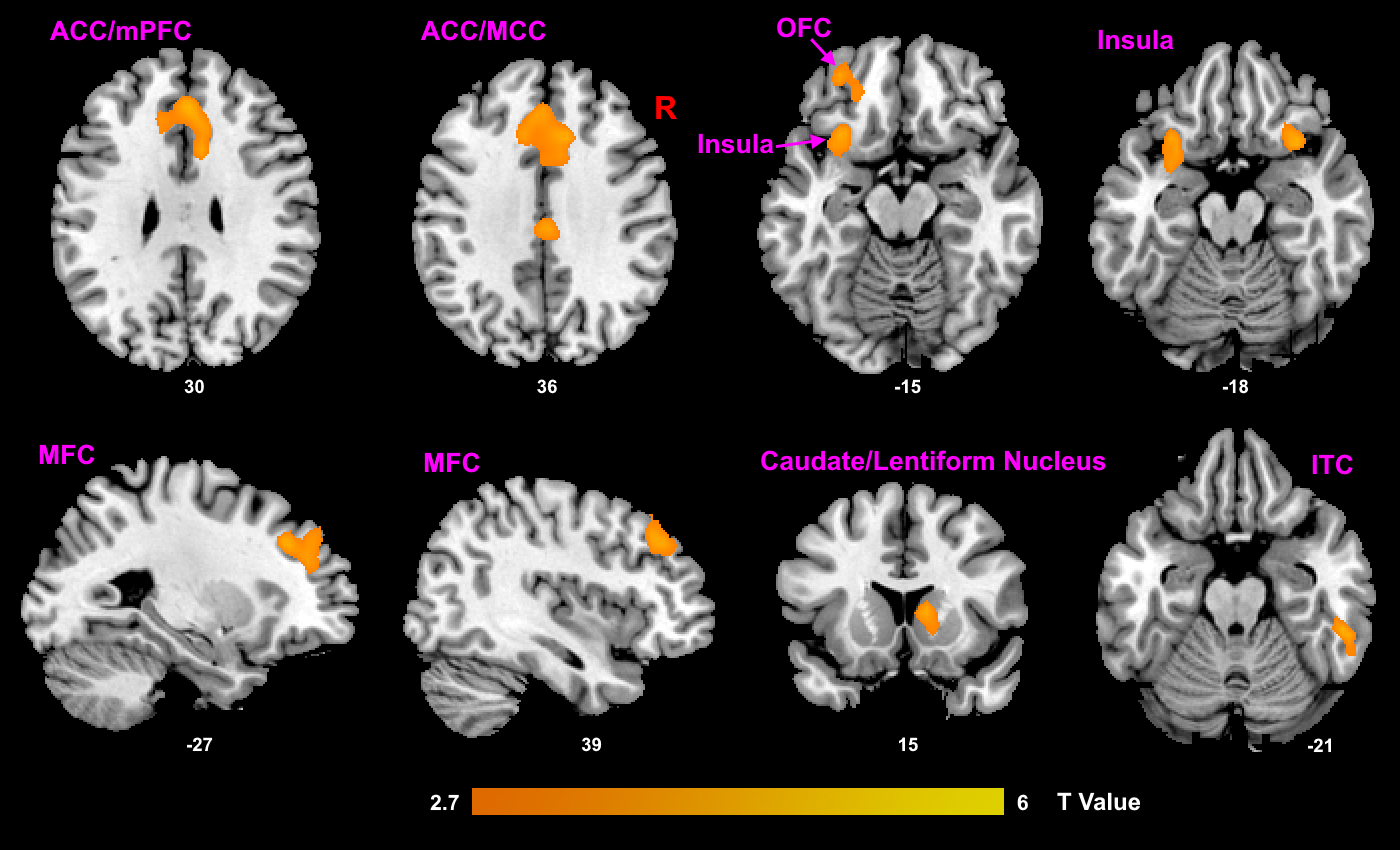


**Figure S3:** Altered posterior hypothalamus-related FC between PMDD patients and HCs. **Abbreviations:** FC, functional connectivity; PMDD, premenstrual dysphoric disorder; HCs, healthy controls; MFC, middle frontal cortex; SFC, superior frontal cortex; PostCG, postcentral gyrus; ITC, inferior temporal cortex.


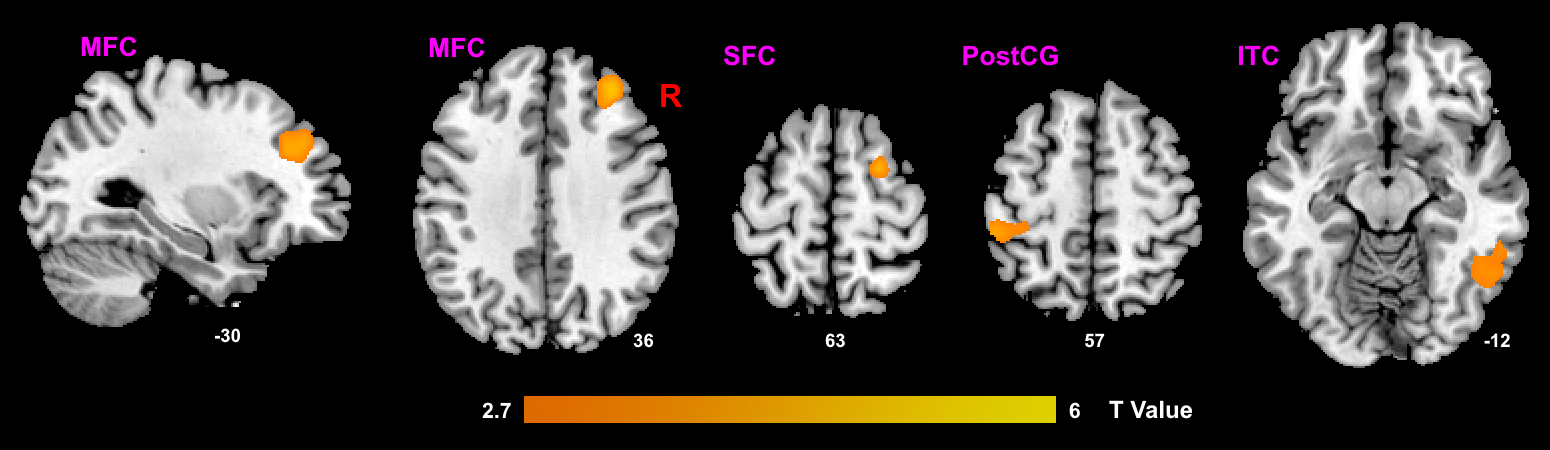

Supplement: Supplementary file 1 — Supporting Information Detailed inclusion criteria for PMDD patients. Hypothalamic subregions‐related FC results. Figure S1: Altered anterior‐superior (A) and anterior‐inferior (B) hypothalamus‐related FC between PMDD patients and HCs. Figure S2: Altered intermediate hypothalamus‐related FC between PMDD patients and HCs. Figure S3: Altered posterior hypothalamus‐related FC between PMDD patients and HCs. [file DA-2026-3641238-s001.docx]
